# Alcoholism and Tuberculosis

**Published:** 1907-10

**Authors:** Louis C. Rouglin

**Affiliations:** Atlanta, Ga.


### ALCOHOLISM AND TUBERCULOSIS.*

Louis C. Rouglin, M. D., Atlanta, Ga.

Not a Prohibition Lecture—Biological study of Alcohol-Secretion and Excretion—Alcohol an Excretion—Influence of Excreta generally on Living Matter—the Food Value of Alcohol—Statistics on Relation of Alcohol to Tuberculosis.

Let me say at the outset that this paper is not intended as a prohibition lecture. I am not one of those who believe that the world would have been more blessed had alcohol never been discovered, nor that prohibitive legislation and Government control injuring self-reliance in the individual, a factor so easily destroyed and so hard to regain, will solve the problem of temperance, as Johnson remarked:

"How small of all that human hearts endure
That part which kings or laws can cure."

Education of the masses of the injurious effects of alcohol, its relation to the disease, suffering and degeneration, bringing before the people records and statistics based not on sentiment but on scientific and undisputed facts, will be a greater factor in prevention of its abuse and consequent suffering it entails than all legislation combined.

ALCOHOL—SOURCE, AND ITS RELATION TO LIVING TISSUES.

Ethyl alcohol is one of the normal products of metabolism of the yeast plant, this plant belongs to the class known as fungi, which is the lowest sub-kingdom of plants, and is distinguished morphologically by absence of root, stem and leaf, and physiologically by the absence of green coloring matter known as chlorophyl. Now, it has been clearly demonstrated that only those organisms which possess chlorophyl are able to build up complex food substances from inorganic compounds, while organisms not possessing chlorophyl are unable to utilize the inert organic materials of their environment and are dependent on the chlorophyl bearing plants for their food. Thus, animals live on cellulose, starches, oils and proteids elaborated by green plants for their own use, while fungi is a simpler but humbler way to subsist on the decaying matter of field and forest, we thus see that the fungus possesses many points in common with animals. In other words, its life energies are liberated from the highly organized foods which it first consumes, then decomposes.

SECRETION AND EXCRETION.

Every living organism absorbs certain food-stuffs, assimilates these, and either directly or indirectly couses their catabolism, which results in the formation of a number of substances of simpler composition, which are passed out by the cell or organism.

Matter passing out of living cells is divided into two classes, (a) Secretion, and (b) Excretion.

(a) A secretion is any substance which is elaborated within the cells and passes out in the surrounding medium where it performs a function or serves a purpose advantageous to the cell or to the organism of which the cell may be a part.

(b) An excretion is any substance which is the product of catabolism of food, from which the organism has extracted the maximum energy possible for it, and which would injure the cells that formed it, if retained in them, and is expelled by the cells immediately after its formation.

### ALCOHOL AN EXCRETION.

Succinic acid, $H_2O$, $CO_2$, glycerine and alcohol a,re among the many substances that leave the yeast plant. No contention has ever been made that the yeast plant makes any use of these substances after they are thrown out of the body; in fact, it throws them out because it has no further use for them, and can get no further energy from them, therefore all these substances are and must be classed as excretions. Thus we see that Ethyl alcohol is a typical excretion of the yeast plant.

### EXCRETA SUB-DIVIDED

Excreta is thrown out for two reasons; first, the organism has no further use for it; second, excreta if retained is injurious to the organism. Vaughan expresses this biological law as follows: "The cells of the body as well as bacteria are injured when the products of their own activity accumulate about them."

Excreta is subdivided into (a) Toxic ptomaine formed in the presence of little oxygen, and (b) non-toxic ptomaine found in the presence of abundant or free oxygen. In his work on Ptomaine, Lecomaines,Toxins, and Anti-Toxins, Vaughan says: "The excretion of all living things, plants and animals, contain substances which are poisonous to the organisms which excrete them. These poisons originate in the metabolic changes by which the complex organic molecule is split up into simpler compounds," and Hall proves that not only is the excreta poisonous to the organism which produces it, but also has a toxic action on any organism of a higher rank.

Now, let us see whether alcohol, the excreta of the yeast plant, is toxic to its producer. Carl Oppenheimer, the greatest authority on fermentation, says, "The question as to how far the cleavage products affect the ferment injuriously, can be answered very easily in the case of alcoholic fermentation, since in this case one of the cleavage products alcohol is, to a certain degree of concentration, a protoplasmic poison and injurious to the yeast and decreases fermentation."

From the above we can now get the following deductions:

1. Fungi have many points in common with animals.

2. Alcohol is the excretion of a fungus—the yeast plant.

3. Excreta toxic to its producer is toxic to all higher organisms.

4. Alcohol, being toxic, to the yeast plant, is therefore according to the universal law, toxic to all higher organisms.

Now, with these relations of alcohol to the living tissue being already set forth, the relation of alcohol to tuberculosis may now be more clearly comprehended.

### ALCOHOL IS NOT A FOOD.

The value of alcohol as a food in consumptives is vaunted from time to time, and has often been the cause of much misery and suffering to its victims, and although alcohol possesses several characteristics in sommon with food, yet it may be said without reservation that in the scientific significance of the word, alcohol is not a food. The following comparisons pointed out by Winfield S. Hall, remain as undisputed facts and is convincing beyond any contention.

| FOOD. | ALCOHOL. |
|---|---|
| 1. A certain quantity will produce a certain effect at first, and the same quantity will always produce the same effect in the healthy body. | 1. A certain quantity will produce a certain effect at first, but it requires more and more to produce the same effect when the drug is used habitually. |
| 2. The habitual use of food never induce san uncontrollable desire for it, in ever increasing amounts. | 2. When used habitually it is likely to produce an uncontrollable desire for more, in ever increasing amounts. |
| 3. After its habitual use a sudden total abstinence never causes any derangement of the central nervous system. | 3. After the habitual use a sudden total abstinence is likely to cause a serious derangement of the central nervous system. |
| 4. Foods are oxidized slowly in the body. | 4. Alcohol is oxidized rapidly in the body. |
| 5. Foods being useful are stored in the body. | 5. Alcohol not being useful is not stored in the body. |
| 6. Foods are the products of constructive activity of protoplasm in the presence of abundant oxygen. | 6. Alcohol is a product of decomposition of food in the presence o. a scarcity of oxygen. |

7. Foods (except meats) are formed in nature for nourishment of living organisms and are, therefore, inherently wholesome.

8. The regular ingestion of food is beneficial to the healthy body, but may be deleterious to the sick.

9. The use of foods is followed by no reaction.

10. The use of food is followed by an increased activity of the muscle cells and brain cells.

11. The use of food is followed by an increase in the excretion of C O 2.

12. The use of food may be followed by an accumulation of fat, notwithstanding increased activity.

13. The use of food is followed by a rise in body temperature.

14. The use of food strengthens and steadies the muscles.

15. The use of food makes the brain more active and accurate.

7. Alçohol is formed in nature only as an excretion. It is, therefore, in common with all excretions, inherently poisonous.

8. The regular ingestion of alcohol is deleterious to the healthy body, but may be beneficial to the sick through its drug action.

9. The use of alcohol in common with narcotics in general, is followed by a reaction.

10. The use of alcohol is followed by a decrease in the activity of the muscle cells and brain cells.

11. The use of alcohol is followed by a decrease in the excretion of C O 2.

12. The use of alcohol is usually followed by an accumulation of fat through decreased activity.

13. The use of alcohol may be followed by a fall in body temperature.

14. The use of alcohol weakens and unsteadies the muscles.

15. The use of alcohol makes the brain less active and less accurate.

STATISTICS SHOWING RELATION OF ALCOHOL TO TUBER-

The following statistics are compiled from reports made by Professor von Burge: In a group of 422 cases in which father, mother and daughter were on the whole abstemious, consumption prevailed as follows: Fathers 3.3 per cent, mothers 2.8 per cent., daughters 1.6 per cent., while in a group of 435 cases where fathers, mothers and daughters used alcohol to excess, consumption prevailed as follows:

Fathers 16.2 per cent., mothers 18.2 per cent., daughters 10.7 per cent.

In another group of 318 cases in which the fathers were total abstainers, or used alcohol moderately, consumption in the offspring prevailed 19.4 per cent., while in 127 cases, where the fathers used alcohol to excess, consumption in the offspring prevailed 38.1 per cent., actually showing that the children of alcoholic parents are 100 per cent. more liable to consumption than those of abstemious parents.

From what has been said above the following may be summarized:

1. Alcohol, the excretion of a fungus (yeast plant) and toxic to its producer, has a deleterious toxic action on organisms of higher rank.

2. Alcohol is not nutritious and has no food value.

3. The offsprings of alcoholic parents are 100 per cent. more liable to consumption than the offspring of abstemious parents.

4. That, as cause and effect, alcohol and consumption are allied to one another, and that a definite relationship exists between alcohol and tuberculosis.

829-830 Candler Building.

